# Plant HDAC inhibitor chrysin arrest cell growth and induce *p21*^*WAF1*^ by altering chromatin of STAT response element in A375 cells

**DOI:** 10.1186/1471-2407-12-180

**Published:** 2012-05-16

**Authors:** Manika Pal-Bhadra, M Janaki Ramaiah, T Lakshminarayan Reddy, Anita Krishnan, SNCVL Pushpavalli, K Suresh Babu, Ashok K Tiwari, J Madhusudana Rao, Jhillu S Yadav, Utpal Bhadra

**Affiliations:** 1 Department of Chemical Biology, Indian Institute of Chemical Technology, Uppal Road, Hyderabad, 500007, India; 2 Functional Genomics and Gene silencing Group, Centre for Cellular and Molecular Biology, Uppal Road, Hyderabad, 500007, India; 3 Department of Natural Product, Indian Institute of Chemical Technology, Uppal Road, Hyderabad, 500007, India; 4 Department of Pharmacology, Indian Institute of Chemical Technology, Uppal Road, Hyderabad, 500007, India

**Keywords:** HDAC-8, Chrysin, A375 cells, HDAC inhibitor, p21^WAF1^, Cell cycle arrest, p21 promoter, STAT, Apoptosis

## Abstract

**Background:**

Chrysin and its analogues, belongs to flavonoid family and possess potential anti-tumour activity. The aim of this study is to determine the molecular mechanism by which chrysin controls cell growth and induce apoptosis in A375 cells.

**Methods:**

Effect of chrysin and its analogues on cell viability and cell cycle analysis was determined by MTT assay and flowcytometry. A series of Western blots was performed to determine the effect of chrysin on important cell cycle regulatory proteins (Cdk2, cyclin D1, p53, p21, p27). The fluorimetry and calorimetry based assays was conducted for characterization of chrysin as HDAC inhibitor. The changes in histone tail modification such as acetylation and methylation was studied after chrysin treatment was estimated by immuno-fluorescence and western blot analysis. The expression of Bcl-xL, survivin and caspase-3 was estimated in chrysin treated cells. The effect of chrysin on p21 promoter activity was studied by luciferase and ChIP assays.

**Results:**

Chrysin cause G1 cell cycle arrest and found to inhibit HDAC-2 and HDAC-8. Chrysin treated cells have shown increase in the levels of H3acK14, H4acK12, H4acK16 and decrease in H3me2K9 methylation. The p21 induction by chrysin treatment was found to be independent of p53 status. The chromatin remodelling at p21^WAF1^ promoter induces p21 activity, increased STAT-1 expression and epigenetic modifications that are responsible for ultimate cell cycle arrest and apoptosis.

**Conclusion:**

Chrysin shows *in vitro* anti-cancer activity that is correlated with induction of histone hyperacetylation and possible recruitment of STAT-1, 3, 5 proteins at STAT (−692 to −684) region of p21 promoter. Our results also support an unexpected action of chrysin on the chromatin organization of *p21*^*WAF1*^ promoter through histone methylation and hyper-acetylation. It proposes previously unknown sequence specific chromatin modulations in the STAT responsive elements for regulating cell cycle progression negatively via the induction of the CDK inhibitor *p21*^*WAF1*^*.*

## Background

Melanoma is the most aggressive skin cancer but is highly resistant to available therapies [1]. Rapid cell proliferation during tumorigenesis is mainly associated with two major events, abnormalities in chromatin structure and functional defects (mutations) in tumor suppressor genes and/or oncogenes [2]. Recently, modifications in the chromatin structure by the loss or gain of DNA methylation and post-translational histone modifications have emerged as important contributors in tumor formation [3]‐[5]. The delicate balance between histone acetyl transferases (HATs) and histone deacetylases (HDACs) regulate the state of histone acetylation [6]. HDACs include HDAC-1, 2, 3 and 8 belongs to Class I that are found in nucleus, HDAC-4, 5, 7 and 9 belongs to class IIa and HDAC-6 and 10 belongs to class IIb and shuttle between nucleus and cytoplasm of the cell [7]. A delicate balance between histone acetyl transferases (HATs) and histone deacetylases at the histone H3 and H4 tails of core nucleosomal complex affects chromatin folding and chromosomal movement required for transcription that leads to normal cell growth. In general histone tails (i.e N terminal tails) are subjected to wide variety of post-translational modifications that includes acetylation, methylation, phosphorylation, ubiquitination, citrullination, ADP-ribosylation and SUMOylation[2]. Such modifications control the ability of the histone tails to interact with key chromatin or transcriptional regulators. Among various modifications, acetylation and deacetylation plays a central role in controlling transcriptional activity in malignant cells [8]‐[10]. Histone acetylation leads to transcriptional activation where as deacetylation leads to transcriptional repression or silencing [11]. The histone acetylating enzymes (HATs) and histone deacetylating enzymes (HDACs) can be targeted to specific regions of the genome results in maintenance of acetylation based epigenetic code. This code will be read by non-histone proteins that bind in an acetylation dependent manner or through direct effects on chromatin structure [10,12]. Studies on HDACs, associated with several oncogenes and tumor suppressor genes indicate the potential involvement of HDACs in tumorigenesis [13,14]. In addition, the pattern of histone acetylation as well as distribution of stable methylation and phosphorylation represents a functional code that is recognized by the non-histone protein complexes involved in the regulation of gene transcription [15]‐[17]. However, their role in tumor formation has not been critically analyzed.

Cell cycle progression is primarily controlled by a family of cyclin-dependent kinases that can be negatively regulated by CDK inhibitor *p21*^*WAF1*^[14]. In many cases, *p21*^*WAF1*^ activity is coupled with the histone acetylation at the promoter level [18]. The novelty of plant chrysin is to delocalize stable histone methylation that corroborates with other histone modifications for blocking rapid cell proliferation in various tumor cells. More over plant flavonoids favour the localized modifications in the chromatin organization at the p21 promoter in tumor cells that are distinct from other HDAC inhibitors such as TSA, SAHA etc. Apart from p21, STAT proteins were found to regulate the transcriptional activation of genes that are involved in cell cycle and cell death such as Bcl-xL, caspases, Fas, TRAIL and p21 [19]. Signal transducers and activators of transcription (STATs) are latent cytoplasmic transcription factors that mediate various responses such as cell proliferation, survival, apoptosis and differentiation. STAT proteins including STAT-1, 3, 5 bind to the DNA and regulate the functions of cell death and cell proliferation respectively [20]. Among the different STAT proteins available in the cell only STAT-1 was found to regulate the process of cell death by transcriptional mechanism involving activation of death promoting genes as well as non-transcriptionally by interacting with TRADD, p53 or HDAC [21].

Chrysin and its analogues are a group of poly phenolic compounds that are found in fruits, vegetables, olive oil, tea and red wine [22]. Plants produce flavonoids as secondary metabolites for protection against micro organisms, U.V.light, spread of disease and gives colour to flowers. Chrysin is 5,7-dihydroxy flavone that was found to be cytotoxic with EC_50_ value of 100 μM in wide range of cell lines such as breast (MCF-7, MDA-MB-231 cells), colon (Lovo, DLD-1) and prostate cancer cells [23,24]. Emerging evidences have shown that Histone deacetylase inhibitors (HDACi) such as Trichostatin A (TSA), NBM-HD-1, 3, 3' Diindolyl methane (DIM) were found to be not only inhibit histone deacetylase activity but also decrease the Akt activity that eventually lead to growth inhibition as well as apoptosis [25]‐[28]. Recent studies have shown the Akt inhibitory activity and apoptotic inducing nature of chrysin [29,30]. But the exact molecular mechanism of action of chrysin was not studied. In the present study we have identified that chrysin functions as HDAC-8 inhibitor and how chrysin controls the cell cycle and cause G1 cell cycle arrest by regulating various cell cycle proteins and histone modifications (H3acK14, H4acK12, H4acK16 and H3K9 me2) at p21 promoter. Here we establish the role of STAT response element (−684/−692) in the transcriptional activity of p21.

## Results

### Isolation, purification and characterization of novel flavonoids

Chrysin (C15H10O4) and its two derivatives, oroxylin-A and methoxy-chrysin (Additional file 1: Figure S1), were extracted from the dried stem bark of the *Oroxylum indicum* plant using petroleum ether extraction and from the soluble fractions of the same extract using acetone (Additional file 1). The identities and structures were established by NMR (Additional file 1: Figure S2, Figure S3) and ESI-MS analyses (Additional file 1: Table S1, Table S3). The identities were verified by comparing the spectroscopic results as described earlier [31]. The compounds were purified further by HPLC. The HPLC fractions that provide greater than 97–99 % level of purity of the compounds were considered further ( Additional file 1: Figure S1).The base structure of all three compounds is flavonoid. In oroxylin-A, one methoxy group (Meo) at 6^th^ position of chrysin, while in methoxy chrysin, the methoxy group substitution at 7^th^ position of chrysin ( Additional file 1: Figure S1). The presence of the voluminous hydrophobic (OH) substitute at the R6 in the chrysin causes an inhibitory effect on DNA cross-linking.

### Role of Chrysin in cell cycle progression

**Figure 1:**
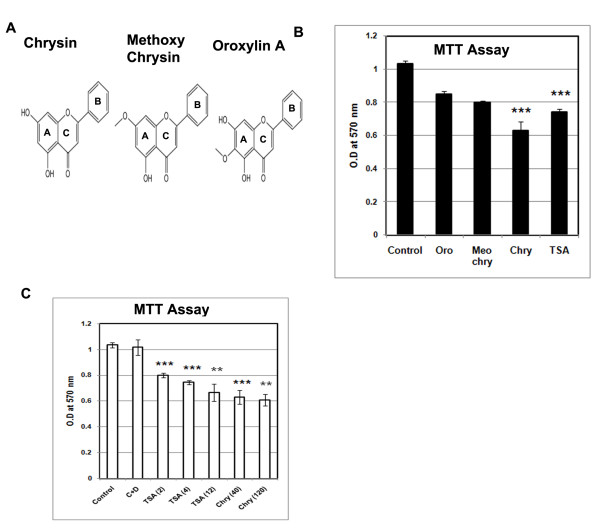
**(A) Chemical structures of Chrysin, Methoxy Chrysin and Oroxylin A. (B)** Chrysin affects cell viability. Human melanoma A375 cells were seeded at density of 10,000 cells per well of 96 well plate and treated with 40 μM of Oroxylin A (Oro), Methoxy Chrysin (Meo Chry), Chrysin (Chry) at a concentration of 40 μM and 4 μM Trichostatin A (TSA) for 24 h. The cell viability of A375 cells was measured by MTT assay 24 h after compound treatment. Data represents mean ± S.D of three independent experiments performed. *** represents P < 0.001; ** represents p < 0.01. **(C)** Comparative studies between chrysin (40 and 120 μM) and TSA (2, 4 and 12 μM) was made and the cell viability was analysed.

The effect of the chrysin and its analogues (Figure 1A) on the cell viability and cell cycle was determined by MTT assay and FACS analysis in human neoplastic A375 cells.The incubation in 40 μM chrysin or its derivatives showed marked inhibition on cell proliferation. Chrysin having 2 hydroxyl groups cause 50 % of cell death at 40 μM concentration. At lower concentration (10 and 20 μM) a trace level of cytotoxic effect was noticed (data not shown). A375 cells treated with analogues of chrysin, oroxylin A and methoxy chrysin show less cytotoxicity (Figure 1B). But cytotoxicity was not increased proportionate to the higher concentration (120 μM) in A375 cell line over 48 h of incubation. We also compare the effect of chrysin with known HDAC inhibitor Trichostatin A to understand the role of chrysin on cell viability relative to standard HDAC inhibitor TSA (Figure 1C).

**Figure 2:**
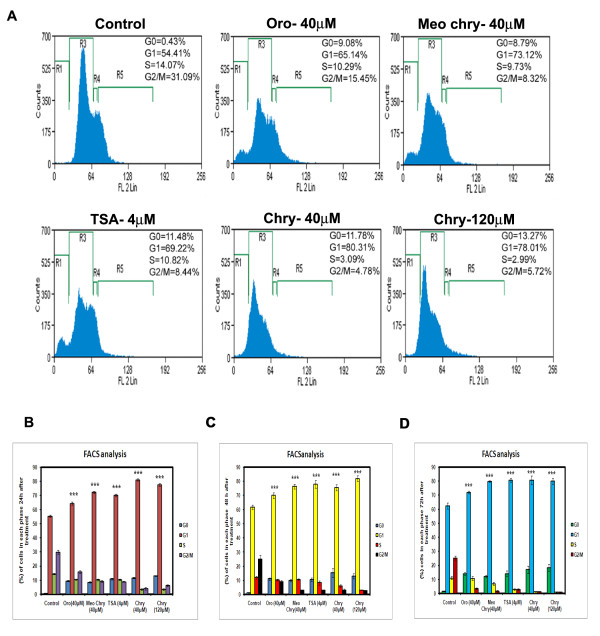
**Chrysin cause G1 cell cycle arrest.(A)** Human melanoma A 375 cells were treated with 40 &120 μM of Chrysin (**Chry**), 40 μM of Oroxylin A (**Oro**), Methoxy-chrysin (**Meo chry**) for 24 h. Cell cycle analysis was performed by flowcytometry after propidium iodide staining. The X-axis (FL-2) shows DNA content, and Y-axis (counts) shows the number of cells presented in each phase. TSA (4 μM) was used as positive control. **(B,C &D)** The data obtained from FACS analysis for a time period of 24, 48 and 72 h was represented in the form of bar diagram. Each experiment was conducted three times and standard deviations were derived. *** indicates p < 0.001, ** indicates p < 0.01, * indicates p < 0.05. The p-values were derived in compound treated cells when compared with control untreated cells using Graphpad software.

In order to understand the regulatory role of chrysin on the cell cycle progression flow cytometric analysis (FACS) was conducted. The A375 cells treated with 40 μM of chrysin showed a strong accumulation of cells (80 %) in the G1 phase relative to 55 % accumulation in the DMSO treated control cells. The same cells incubated at 4 μM of TSA caused 69 % G1 arrest. Chrysin analogues such as oroxylin A and methoxy chrysin have shown 65 % and 73 % of cells accumulated in G1 phase (Figure 2A) when cells were incubated at 24 h. In longer incubation time (48 and 72 h) cells showed an increase in G0 (apoptotic cells) and G1 phase cells (Figure 2B–D). Therefore, chrysin and its analogues arrest A375 melanoma cells at the G1 phase.

### Chrysin as histone deacetylase inhibitor

**Figure 3:**
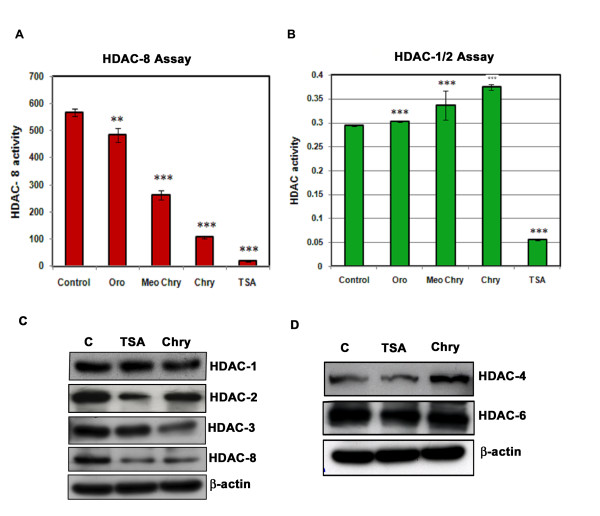
**Chrysin inhibits HDACs.** Histone deacetylase 8 activity **(A)** and histone deacetylase 1/2 activity **(B)** was measured with compounds Chrysin (**Chry**), Oroxylin A (**Oro**) and methoxy chrysin (**Meo Chry**) at 40 μM final concentration and TSA at 4 μM. The data from three independent experiments was depicted in bar diagram. Each experiment was repeated three times. *** represents P < 0.001; ** represents p < 0.01 **(C&D)** Western blot analysis of class I (HDAC-1, 2, 3, 8) and Class II (HDAC −4, 6) proteins was performed in cells treated with Chrysin (Chry) and TSA at 40 μM and 4 μM concentrations for 24 h respectively. Chrysin was shown to decrease the HDAC-2, 3, 8 protein level.

It was shown earlier that HDACs 1, 2, 3 and 8 are referred as class I type where as HDACs 4, 5, 6, 7, 9 and 10 are known as class II type HDACs [8,32]. To characterize chrysin as reliable and potential histone deacetylase (HDAC) inhibitor, the effect of chrysin and a known HDAC inhibitor Trichostatin A (TSA) on the HDAC- 8 activity was compared by *in vitro* HDAC enzymatic assay. Chrysin and TSA inhibit histone deacetylase 8 (HDAC-8) activities strongly (Figure 3A). Conversely, the reduction in HDAC- 8 activity was relatively less in chrysin analogues such as oroxylin A and methoxy-chrysin suggesting that chrysin as a potent HDAC-8 inhibitor. To further confirm the inhibitory action of chrysin on HDAC-8 we carried out HDAC1/2 assay. We did not observe any change in the activity of HDAC1/2 upon addition of chrysin (Figure 3B).

To measure the effect of chrysin and TSA on protein levels of HDACs a series of western blot analyses was performed using cell lysate extracted from treated A375 cells. The levels of HDAC-2, 3 and 8 proteins were significantly decreased by treatment of chrysin (40 μM) or TSA (4 μM) (Figure 3C). However their effect is less pronounced in HDAC-1 protein. In contrast , cells treated with chrysin and TSA did not show significant effect on HDAC-4 and 6 protein levels relative to control (C) untreated cells (Figure 3C &3D). Therefore chrysin functions as HDAC-2 & 8 inhibitor. Similarly apigenin, a flavonoid and a close analog of chrysin was found to inhibt the histone deacetyalse activity [33].

### Regulation of cell cycle components

**Figure 4:**
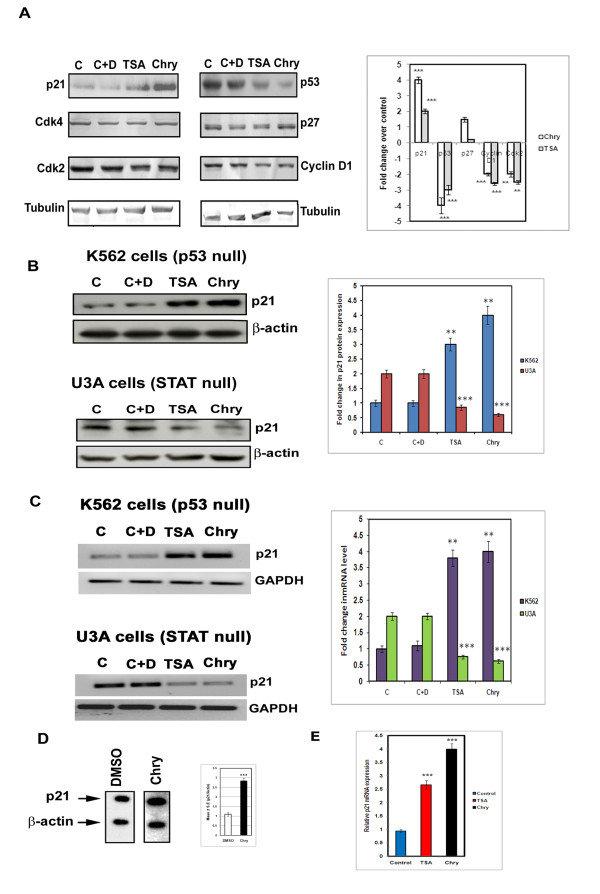
**Effect of chrysin on proteins that regulate cell cycle.(A)** The effect of chrysin on Cdk4, Cdk2, Cyclin D1, p53, p21 and p27 proteins were determined by Western blot analysis from the total protein extracted from A375 cells after incubating with 40 μM of Chrysin and 4 μM of TSA for a time period of 24 h. The blots were reprobed with Tubulin antibody that acts as gel loading control. C is the control untreated cells. C + D: is the control cells treated with DMSO (0.1 %). Bar diagram representing the intensity of expression of p21, p53, p27, cyclin D1, Cdk2 proteins. **(B)** The induction of p21 protein by compounds Chrysin 40 μM(**Chry**) and TSA 4 μM was studied by western blot analysis in K562 leukemia cells which is null for p53 and U3A, fibrosarcoma cells which is null for STAT-1 . We observed induction of p21 was independent of p53 and dependent on STAT-1. C: Control, untreated cells and C + D represents control untreated cells incubated with 0.1 % DMSO. Each experiment was conducted three times and standard deviations were derived. Data represents mean ± S.D of three independent experiments performed. _***_ represents P < 0.001; ** represents p < 0.01. **(C)** The effect of chrysin on mRNAlevels of p21 in K562 (p53−/−) and U3A (STAT1 −/−) cells. P21 induction was observed 3–4 folds in chrysin (40 μM) treated K562 cells but not in U3A cells which lack STAT-1 . These treatments were carried out for 24 h **(D)** Transcriptional run-on assays using radio-labeled nuclear RNA extracted from 0.1 % DMSO and chrysin (40 μM) treated A375 cells after 24 hrs. Actin nuclear RNA was used as internal control. The relative ratio of P21/ Actin from triplicate blots were shown in a bar chart. _***_ represents P < 0.001; ** represents p < 0.01. **(E)** The quantitative real-time PCR assay for the p21 mRNA in chrysin (40 μM) and TSA (4 μM) treated A375 cells for 24 h. Control indicates cells treated with DMSO (0.1 %).

Previous studies have established that deacetylation of histones by HDAC enzymes cause inactivation of tumor suppressor genes leading to neoplastic transformation [34] Inhibition of HDAC enzymatic activity restores the expression of many tumor suppressor genes. Thus the amount of tumor suppressor proteins *p53, p27* and *p21*^*WAF1*^ was estimated from the cell lysates of A375 neoplastic cells incubated with 0.1 % DMSO, chrysin and TSA containing media. The *p21*^*WAF1*^ protein in A375 cells was increased 4-folds in 40 μM chrysin, while there was no significant change in the level of p27 protein. The expression of tumor suppressor protein *p53* was drastically reduced in chrysin treated cells when compared to control untreated cells (Figure 4A). Trichostatin A, a class I and class II HDAC inhibitor has shown similar effect on mRNA and protein levels of p21 and p53 [1]. Apigenin, a flavonoid caused p21 induction with the increase of p53 in 22Rv1 cells and the same compound caused p21 induction in PC-3 prostate cancer cells which lacks p53. Thus p21 induction is cell type dependent and is independent of p53 status [33].

P21 is a cdk inhibitor whose activation leads to cell cycle arrest or apoptosis [35]. Although the role of p21 in apoptosis is controversial, the HDAC inhibitor sodium butyrate has induced apoptosis in MCF-7 breast cancer cells. Thus acts as a modulator of apoptosis [36]. As cyclin-dependent kinases (Cdks) and cyclins are required for the complex formation with *p21*^*WAF1*^, the level of Cdk2, Cdk4 and Cyclin D1, that regulate G1-S phase transition was also estimated in chrysin (40 μM) and TSA (4 μM) treated cells (Figure 4A). Reduction in cyclin D1 and cdk2 protein level with no change in cdk4 was noticed. To confirm the importance or dependency of p21 induction on p53 and STAT-1 status A375 cells were treated with chrysin (40 μM) and TSA (40 μM) in K562 (p53 null) [37] and U3A ( STAT-1 null) cells for 24 h and conducted western and RT-PCR analysis. We observed pronounced increase (3–4 folds) of both mRNA and protein level of p21 in case of K562 cells and drastic reduction in case of U3A cells. This shows the functional dependency of p21 on STAT-1 protein than p53 protein. Similar results of STAT-1 dependency, p53-independent expression of p21 modulating apoptosis was observed in oxysterol compound treated cells [38] (Figure 4B4C &4D).

To further confirm whether *p21*^*WAF1*^ induction occurs at the transcriptional and post-transcriptional level, nuclei were prepared from the cells incubated in 40 μM chrysin or 0.1 % DMSO containing culture media. The results of the nuclear run-on experiments showed that the amount of nascent *p21*^*WAF1*^ transcripts was increased more than 2.5-fold (Figure 4D). To further confirm the expression of p21 mRNA in chrysin treated A375 cells quantitative real-time PCR analysis was conducted and there was upto 4-fold increase in the p21 mRNA level in chrysin treated cells (Figure 4E).

### Dual post-translational histone tail modifications

**Figure 5:**
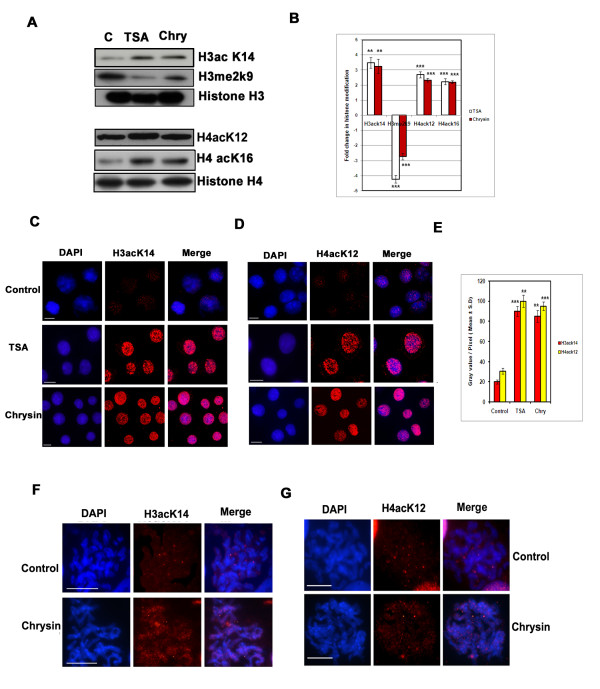
**Effect of chrysin on histone acetylation and methylation.(A)** Histone protein expression pattern with respect to acetylation and methylation in the chrysin (40 μM) treated cells The amount of histone H3acK14, H4acK12, H4acK16 and histone H3 dimethyl lysine 9 are measured by Western blot analysis from A375 cells treated with DMSO, chrysin (40 μM) and TSA (4 μM) for 24 h. The blots are re-probed with total histone H3 and histone H4 proteins for gel loading control. **(B)** Bar diagrams representing the relative ratio (mean ratios ± S.D) between histone modifications upon chrysin and TSA treatment and were calculated from three independent experiments. *** represents P < 0.001; ** represents p < 0.01. **(C&D)** Human melanoma A 375 cells were treated with chrysin at 40 μM concentration and TSA at 4 μM concentration and the nuclei were subjected to immunostaining using H3acK14, H4acK12 antibodies. **(E)** Quantitative estimation of histone modifications (H3acK14 and H4acK12) in interphase nuclei in TSA and chrysin treated cells. Histogram represents quantitative measurement of histone H3acK14 and H4acK12 status as fluorescence intensity. **(F&G)** Metaphase spreads isolated from pre-incubated A375 cells with chrysin 40 μM and control untreated cells hybridized with histone H3acK14 and H4acK12 antibodies. Corresponding DAPI stained images of the same metaphase spreads are shown. Scale-10 μm.

HDACs are the major proteins that control the nucleosome conformation and chromatin organization. To identify causal factors for induction of *p21*^*WAF1*^, distinct chromatin modifications such as, acetylated histone H3 and H4 proteins were assayed from the A375 cells that are incubated in different compounds (0.1 % DMSO, 40 μM chrysin and 4 μM TSA) for 24 h. Western blot analyses was carried out by probing with acetylated histone [ H3 Lysine 14, H4 Lysine 12 ,H4 Lysine 16] and histone H3 and H4 antibodies. Culture of the A375 cells in chrysin and TSA containing media induced acetylated histone (i.e H3acK14, H4acK12, H4acK16) levels markedly (Figure 5A, 5B). Therefore typical to HDAC inhibitors, chrysin improves acelylated lysine levels of histone H3 and H4 tails in A375 tumor cells. Incubation in chrysin (40 μM) and TSA (4 μM) reduces methylation signals (H3me2K9) by 3-folds. These findings demonstrate that chrysin-dependent modulation of acetylation and methylation of histone (H3 and H4) lysine residues form a functional complex that might add the epigenetic marks on the chromatin structure required for blocking rapid cell proliferation (Figure 5A, 5B).

### Histone tail modification in inter-phase nuclei and distribution of histone modifiers in the metaphase chromosomes

To visualize the accumulation of histone acetylation in the interphase nuclei, A375 cells were treated either with 0.1 % DMSO, chrysin (40 μM), TSA (4 μM) separately for 24 h and processed for indirect immuno-fluorescence using histone H3acK14 and H4acK12 antibodies. Increase in the acetylation of histone H3acK14 and H4acK12 in the A375 cell nuclei was observed (Figure 5C–5E). The increased acetylated H3K14 and H4K12 by chrysin was strongly correlated with the cell cycle arrest and *p21*^*WAF1*^ induction. Similar results were also observed in metaphase spreads with respect to acetylation pattern of histones H3 and H4 (i.e H3ack14 & H4ack12) (Figure 5F, 5G).

**Figure 6:**
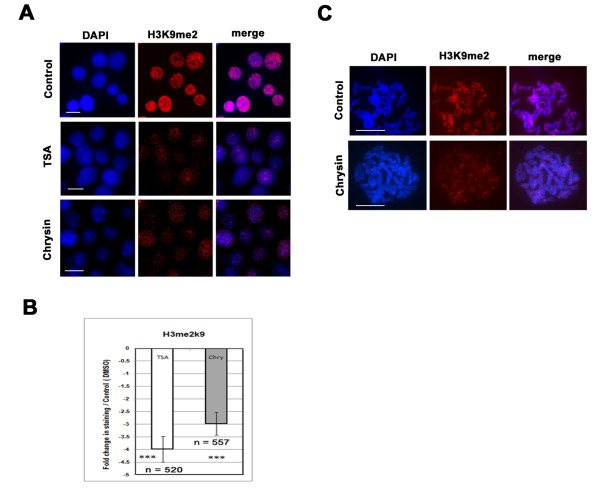
**Chrysin delocalizes methylated histone lysine 9 foci in the interphase nuclei and metaphase chromosomes in A375 cells.** Nuclei and chromosomes were prepared from A375 cells cultured in 0.1 % DMSO, 40 μM chrysin, and TSA (4 μM) for 24 h. **(A)** Interphase nuclei were immunostained with H3me2K9 foci in counterstained with DAPI. Merge figures are shown in the panel. Scale-10 μm **(B)** Bar diagram shows amount of histone H3me2K9 antibody hybridized with interphase nuclei based on fluorescence intensity. Number of nuclei (n) in each cell is noted below. *** represents P < 0.001. **(C)** Metaphase chromosomes immunostained with H3me2K9 antibody. The chromosomes were prepared from DMSO treated and 40 μM chrysin A375 cells for 24 hrs.

Further we have focussed on pattern of histone methylation in chrysin and TSA treated cells. Incubation with chrysin (40 μM) and TSA (4 μM) showed a clear reduction in the number of histone H3me2K9 foci (Figure 6A). A statistical profile demonstrated that chrysin increased histone H3 and H4 acetylation uniformly in the interphase nuclei of the cancer A375 cells, but decreases lysine9 methylated H3 proteins in the same nuclei (Figure 6B). Our analysis also showed that the distribution of H3me2K9 foci on the metaphase chromosomes isolated from DMSO treated A375 cells (control cells) was more intense than the chrysin exposed cells (Figure 6C). However, no apparent changes in the distribution of foci at the chromocentre were noticed when cells were exposed to chrysin and control DMSO. Therefore, the loss of histone H3me2K9 is mostly limited to the euchromatic domains. Conversely, a greater accumulation of acetylated histone signals reveals that chrysin might be required for chromatin organization changes for arresting the rapid cell growth. Thus differential distribution of acetylated and methylated lysines by chrysin on the different chromosomal locations marked functional distinction of chromatin organization.

### Accumulation of acetylated histone and displacement from p21^WAF1^ promoter

**Figure 7:**
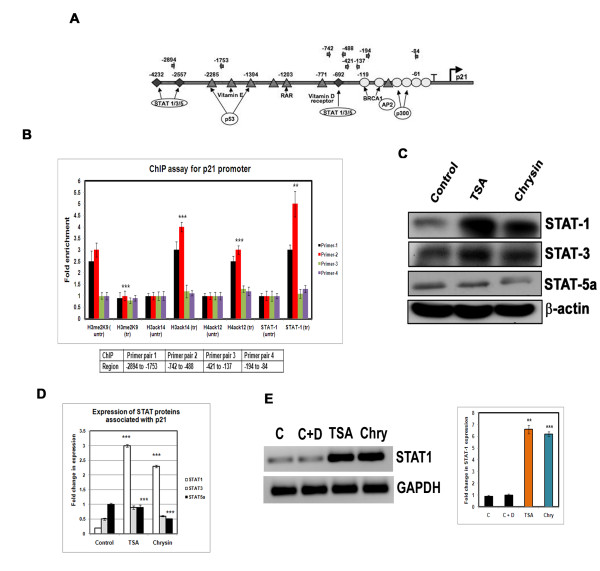
**Effect of chrysin on histone modifications and STAT-1 at p21 promoter.(A)** Schematic diagram of the human *p21*^*WAF1*^ promoter illustrating regulatory factor binding sites like −119 (BRCA1), -692 (STAT-1/3/5), -1203 ( RAR), -1394 and −2285 (P53RE), -4232 and −2557 are the (STAT1/3/5) binding sites.The arrow indicates transcriptional start site at +1. **(B)** Four different regions (−194/-84, -421/-137, -742/-488, -2894/-1753) were amplified by quantitative real time PCR after chromatin immunoprecipitation to observe the region that is highly sensitive to the treatment with chrysin in A375 cells using anti-H3acK14, H4acK12, H3me2K9 and STAT1 antibodies. untr: untreated cells, tr: treated cells.Table below the diagram shows relative location of the four primer pairs used in ChIP assay. **(C)** Effect of chrysin on the expression of p21 associated STAT proteins in Chrysin and TSA treated A375 cells (i.e STAT-1, STAT-3 and STAT-5a). **(D)** Graphical representation of STAT protein expression that is associated with p21 in chrysin (40 μM) and TSA (4 μM) treated cells for 24 h time period. *** represents P < 0.001. **(E)** A375 cells were treated with Chrysin (40 μM) and TSA (4 μM) for 24 h and mRNA was isolated and checked for STAT-1 expression. *** indicates p < 0.001, ** indicates P < 0.01.

To correlate *p21*^*WAF1*^ induction and localized chromatin organization, the binding of acetylated and methylated histone H3 and H4 were analysed at the *p21*^*WAF1*^ promoter using ChIP assay. Since induction of *p21*^*WAF1*^ expression occured independent of *p53* and other members of the Cdk family, we had selected four distinct regions of the *p21*^*WAF1*^ promoter including two STAT binding sites (Figure 7A) [39]. Chromatin immuno precipitation assay (ChIP) was conducted to know the effect of chrysin (40 μM) on p21 promoter with regard to STAT1 and histone proteins is concerned. The DNA from the immunoprecipitated chromatin of the A375 cells after incubating in chrysin (40 μM) for 24 hrs was amplified by the quantitative Real-time PCR. The amplified proximal region (−194 to −84) by primer 4 carries core promoter region including the TATA box, the SP1/SP3 binding sites, the E boxes and Ap2 sites [39]. No change in the histone modification was observed (Figure 7B). The second selected region (−421 to −137) amplified by the primer pair 3, which does not contain any typical binding sites represent a non-specific region of *p21*^*WAF1*^ promoter. The histone proteins are almost uniformly distributed with no trace of STAT-1 protein. However, two different amplified fragments (−742 to −488 and −2894 to −1753) carrying STAT protein binding site (TTCNNNGAA) showed a marked enrichment of the acetylated histones H3 and H4 (i.e H3acK14, H4acK12). On the contrary, H3 Lysine 9 methylation on the same regulatory region was reduced in the chrysin treated cells relative to the control untreated cells (untr). The acetylated histones H3ack14, H4ack12 was increased to 3–4 folds in chrysin treated cells. While histone H3k9 methylation showed a profound reduction upto 3-folds in chrysin treated cells in region −742 to −488. Whereas 3-fold increase in H3ack14, 2.5-folds increase in H3ack12 and 2.5-fold decrease in H3k9 methylation in −2894 to −1753 region was observed. The STAT-1 protein levels were increased in both the regions (ie. −742 to −488, −2894 to −1753) (Figure 7B).

Therefore histone tail modifications at the STAT response element are required for *p21*^*WAF1*^ induction and might serve as a switch for *p21*^*WAF1*^ induction by controlling histone modifications [39]. To further confirm the greater accumulation of STAT-1 protein occurred by chrysin exposure, p21 protein was immunoprecipitated and is followed by western blot analyses using STAT-1, 3 and 5 antibodies. Both STAT-1, 3 proteins were increased at an equal level after TSA and chrysin treatment where as STAT-5a was found to be decreased. Probably the ratio of STAT1 and 3 might regulate the cell death event (Figure 7C7D). We have also conducted RT-PCR experiment to study the change in the STAT-1 mRNA level in chrysin (40 μM) treated cells. We found an increase in STAT-1 mRNA level in chrysin treated cells (Figure 7E).

The acetylated histone pattern was increased 2.5 to 3-folds by the incubation in chrysin and TSA containing media, while histone H3K9 methylation showed a profound reduction (4–6 folds) in 40 μM chrysin and 4 μM TSA treated cells. Conversely no changes in the histone acetylation and methylation levels were detected in the p27 promoter by the chrysin incubation (data not shown).

### STAT response element (−692/-684) is important for chrysin mediated *p21*^*WAF1*^ promoter activity

**Figure 8:**
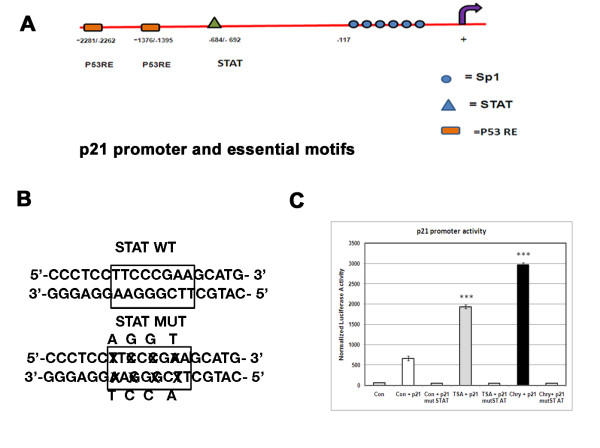
**Effect of chrysin on*p21***^***WAF1***^**promoter acivity and role STAT response element in A375 cells.(A)** The representation of p21 promoter with essential motifs such as 6 Sp1 sites (from −1 to −119), STAT binding site (−692/-684), p53 response element (p53 RE) -1376/-1395, -2281/-2262. **(B)** Representation of wild-type STAT response element (5’-TTCCCGAA-3’) and mutant STAT response element (5’-ATGCGGTA-3’).**(C)** Graph representing p21 promoter activity in A375 cells transfected with p21 promoter which is followed by treatment with chrysin (40 μM) and TSA (4 μM) for 24 h. Chrysin treated cells showed 5–6 fold increase in promoter activity. TSA treated cells showed only 3-folds increase in promoter activity as observed by normalized luciferase values. β-galactosidase O.D values were used for normalizing the Luciferase values.

ChIP analyses reveal that STAT binding site of the *p21*^*WAF1*^ promoter is critical for p21 induction by chrysin (Figure 8A). Thus A375 cells were transfected with 1 μg of p21 promoter (p21-luc) and 500 ng of CMV-β-galactosidase followed by incubation in 0.1 % DMSO, 40 μM chrysin and 4 μM TSA for 24 h and assayed the luciferase activity. β-galactosidase values obtained were used for normalization of luciferase activity. Here DMSO treated cells was used as control. TSA incubated cells have shown 3-folds of *p21*^*WAF1*^ promoter activity whereas chrysin treatment caused a marked increase (6-folds) in promoter activity (Figure 8B, 8C). But the activity was reached to basal level in cells transfected with STAT mutated p21 construct followed by chrysin (40 μM) and TSA (4 μM) treatment.

The mutation of the STAT site fail to activate the chrysin induced *p21*^*WAF1*^ activity, which suggests STAT response element at −692 to −684 is critical for chrysin mediated transactivation of *p21*^*WAF1*^ promoter. It indicates that chrysin is capable of activating p21 transcription through the promoter element in the region −742 to −488 bp containing STAT1/3/5 binding site.

### Effect of chrysin on apoptosis

**Figure 9:**
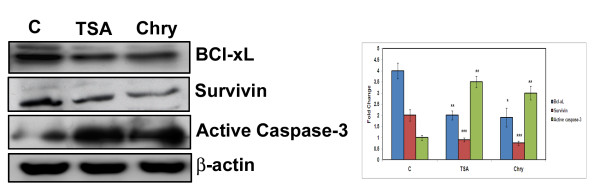
**Chrysin induce apoptosis in A375 cells.** Human melanoma cells. (A375 cells) were treated with TSA (4 μM) and Chrysin (40 μM) for 72 h. The cell lysates were subjected to Westernblot analysis with antibodies against Bcl-xL, survivin and active caspase-3. Each experiment was conducted three times. *** indicates p < 0.001, ** indicates p < 0.01 * indicates p < 0.05. P values were generated in compound [Chrysin (40 μM), TSA (4 μM) ] treated cells when compared to control cells using graph pad soft ware.

STAT-1 and p21 are essential proteins that are involved in modulation and regulation of apoptotic process [36,38]. Recent studies have also focussed on HDAC inhibitors and their repressive role on NF-kB dependent genes i.e Bcl-xL, Survivin [40] to control cell proliferation. Thus we have treated A375 cells with chrysin (40 μM) and TSA (4 μM) for 72 h and lysates were subjected to western blot analyses. We observed a drastic decrease in the levels of anti-apoptotic proteins such as Bcl-xL (a Bcl-2 family protein) and survivin. Interestingly an increase in the level of effector caspase (caspase-3) was also observed. Thus chrysin has a potential role in causing apoptosis (Figure 9).

## Discussion

Cancer is caused by abnormal cell cycle progression. Mammalian cell cycle progression involves the activities of cyclins and cdks. The balance between the activation and inhibition of cyclin/ Cdk inhibitor proteins decide whether cell will proceed through cell cycle or cause cancer [41,42]. Chrysin caused 50 % cytotoxicity than other analogs and this result is in corroboration with the fact that only two hydroxyl groups in AC rings of flavonoid were responsible for effective cytotoxicity. Increase or decrease of -OH groups in flavonoids would lead to loss of potent cytotoxicity [23]. Flowcytometric analysis with chrysin treatment caused an increase in G1 phase cells with concomitant decrease in the number of S and G2/M phase cells, thus confirming G1 cell cycle arrest nature of the compound (Figure 2). Increased time duration of incubation lead to increase of G0 and G1 phase cells. Similar to chrysin, the other HDAC inhibtors such as SAHA and NaB (sodium butyarte) cause G1 cell cycle arrest in neuronal stem cells [43].

Recent developments revealed that HDAC inhibitors are gaining interest as potential anti-cancer drugs due to their ability to reactivate epigenetically silenced genes in cancerous cells and there by control growth arrest, apoptosis and differentiation [44]. The HDAC activity within the cells can be altered by direct inhibition of HDAC enzyme as well as changes in HDAC protein expression. We found decrease in the level of HDAC-8 protein as well as enzyme activity in the chrysin treated melanoma cells (neoplastic) (Figure 3). Similarly decrease in activity and protein levels of HDACs was observed in the case of recent studies on HDAC inhibitors such as Allyl mercaptan (AM), NBM-HD-1, apigenin [26,33,45]. It is a well established concept that HDAC inhibitors induce apoptotic response in a P53-dependent and independent ways [46]. In our study we have observed induction of p21 protein and mRNA in A375 cells with drastic reduction in the p53 protein, level indicating chrysin mediated p21 induction is independent of p53 status in A375 melanoma cells. The p53 independent induction of p21 activity was observed in studies on TSA and Apigenin, a well known flavonoid. This mechanism was reported to be cell type dependent (Figure 4) [1,33].

Typical to HDAC inhibitor, chrysin and its analogues can arrest cell growth and induce *p21*^*WAF1*^ transcription [47-49], but its mechanism of action is quite different from known HDAC inhibitors (TSA). It selectively enhances the accumulation of acetylated histones and STAT proteins at the STAT binding site of the *p21*^*WAF1*^ promoter (Figure 5). Indeed, the novelty of the plant chrysin is to delocalize methyl group from the histone H3 lysine 9 from the STAT response element (−692 to −684) (Figure 6). Reduced histone methylation by treatment with chrysin acts sequentially or in concert with the elevated histone acetylation that might form a complex “histone code”. Such fine-tuning in the chromatin structure precisely in STAT responsive sequence might recognize the non-histone proteins for the transcriptional activation of cdk-inhibitor *p21*^*WAF1*^ gene [15,16,50].

Further, the modulations of histone methylation and acetylation by chrysin might initiate several levels of chromatin modification in the multiple sites such as −684 to −692, −2549 to −2557 required for transcriptional regulation of p21 gene [51]. The histone methylation functions to regulate the chromatin organization directly by affecting higher order packaging of chromatin fiber and is required for the gene transcription and DNA repair mechanism by changing the accessibility of DNA to several transcriptional factors [52,53]. It is known that histone lysine methylation of H3k4 is associated with promoters of actively transcribed genes [54] where as H3K9 lysine methylation is associated with heterochromatin formation [55]. Jumonji-C (jmjC) domain containing enzymes constitute the largest class of histone demthylases which includes JMJD2c and LSD1 and is linked particularly in prostate cancer [56]. Thus we propose that histone tail modifications by the plant chrysin such as methylation and acetylation of lysine are the prominent epigenetic marks that regulate the binding of different transcriptional factors [57-59]. Consistent with this notion, histone modification will allow the recruitment of STAT family of proteins at STAT binding sites in the *p21*^*WAF1*^ promoter [60,61].

The mode of action of chrysin is distinct from the known HDAC inhibitors such as SAHA and TSA. Treatment of SAHA and TSA inhibits LSD1, the known histone lysine demethylase I which demethylate both mono as well as dimethyl lysine 4 of histone H3 that lead to the chromatin modification at the *p21*^*WAF1*^ promoter [62]. But function of chrysin is unique and novel from known HDAC inhibitors which decrease the H3k9 dimethylation at the p21^WAF1^ promoter.

Emerging evidence has indicated p53 independent transcriptional activation of p21 include STAT1, MyoD1 and BRCA1 [63]. Precisely, this study also shows a new regulatory relationship between *p21*^*WAF1*^ and STAT proteins via epigenetic modulation [64]. The changes in the histone code of the chromatin in or near STAT binding sites by the chrysin can increase accessibility of the STAT-1& 3 proteins that lead to activate STAT mediated induction of *p21*^*WAF1*^ expression (Figure 7). Earlier studies indicated the involvement of STAT-1 dependent and p53-independent expression of p21 controlling apoptosis [38]. These results not only suggest that chromatin remodeling within the STAT responsive sites can control transcriptional regulation but also demonstrate that modification in core histone tails by chrysin might activate STAT signals in A375 cells. STAT activated signals in response to IFN-gamma are directly involved in regulating *p21*^*WAF1*^ expression [65]. Nevertheless our findings led to propose a chrysin based novel epigenetic pathway of *p21*^*WAF1*^ regulation by which an increased recruitment of STAT-1and-3 to proximal responsive region from the transcriptional start site in the *p21* promoter that maintain a pivotal role in the *p21*^*WAF1*^ up regulation. We speculate that some unknown binding factors may form a complex with STAT1/3/5 proteins *in vivo* in the presence of chrysin to facilitate STAT1, 3 & 5 for easy recognition and accessibility to the two STAT binding sites. It could be very interesting to identify such chrysin-regulated proteins that bind to STAT binding sites.

In fact, our studies indicate that modification of chromatin structure in response to histone acetylation and methylation of the two responsive sites is sufficient to allow the transcriptional activation of *p21*^*WAF1*^ presumably via STAT proteins (Figure 8). These findings demonstrate a possible working model of chrysin for not only regulating cell cycle but also connect epigenetic modulation of *p21*^*WAF1*^ promoter and STAT signaling pathway as well. The functional importance of STAT region in the promoter activation was highly elucidated. In this study we found that chrysin treatment caused decrease in the protein level of NF-kB dependent genes such as Bcl-xL, survivin that lead to cell death (apoptosis) by enhancing the activity of caspase-3.Thus chrysin can be used as a single drug when compared with combinatorial therapy such as recently used HDAC inhibitor and demethylating agent (Aza Cytidine).

## Conclusions

In summary, we have shown that chrysin posses potent *invitro* anti-cancer activity by suppressing cell proliferation, inducing G1 cell cycle arrest with the upregulation of p21 and decrease in cyclin D1, cdk2 protein levels. This compound caused inhibition of HDAC-8 activity with no effect on the activity of HDAC-1/2. The protein levels of HDAC- 2, 3 and 8 (Class I HDACs) were found to be drastically reduced with no change in HDAC- 4 & 6 (class II HDACs) upon chrysin treatment. Chrysin caused histone modifications such as acetylation and methylation at p21 promoter particularly at STAT binding site (−692/−684) and resulted in increased p21 promoter activity. More over chrysin as a HDAC inhibitor cause apoptosis by decreasing the levels of NF-kB targeted and HDACi related genes such as Bcl-xL , survivin and increased the level of caspase-3 proteins.

## Methods

### Chemical structure and extraction of natural compounds

The dried stem bark of dundilum tree, *Oroxylum indicum* was grinded and extracted consecutively with hexane in a soxhlet apparatus. Solid residue (2.5 g) in the hexane extract was filtered and subjected to silica gel (60–120 mesh) column chromatography to isolate two major fractions (F1 & F2). Fraction F1 was purified on silica gel column chromatography (60–120) eluted with 0.5 % MeoH in Chloroform to isolate methoxy chrysin (0.25 g). Similarly, Fraction F2 was subjected to repeated column chromatography with the elution of 2 % MeoH in Chloroform to isolate oroxylin A (1.2 g) and chrysin (0.8 g). The purification, chemical structure and characterization of all three compounds were determined via extensive spectroscopic NMR, ESI-MS, and HPLC methods. The conserved methyl oxide and hydroxyl group are shown in the chemical structure of small flavonoid compounds.

### Cell culture

A375 ( human melanoma), U3A (Fibrosarcoma) cell lines were maintained in DMEM ( Dulbecco’s Modified Eagle’s Medium). Whereas K562 ( human Leukemia) cell line was maintained in RPMI media. All three cell lines were supplemented with 10 % FCS, 1 % pencillin/ streptomycin & 5 % glutamine. These cell lines were grown at 37^0^ C in a humidified chamber containing 5 % CO_2_.

### MTT assay

Cell viability was assessed by the MTT assay, a mitochondrial function assay. It is based on the ability of viable cells to reduce the MTT to insoluble formazan crystals by mitochondrial dehydrogenase. A375 cells were seeded in a 96-well plate at a density 10,000 cells/well. After overnight incubation, cells were treated with compounds chrysin, methoxy chrysin, oroxylin A at a final concentration of 40 μM and Trichostatin A (TSA) at a final concentration of 4 μM and incubated for 24 h. Medium was then discarded and replaced with 10 μL MTT dye. Plates were incubated at 37°C for 2 h. The resulting formazan crystals were solubilized in 100 μL extraction buffer. The optical density (O.D) was read at 570 using micro plate reader (Multimode Varioskan Flash Instrument-Themo Scientific Ltd).

### Cell Cycle Analysis

5 X 10^5^ A375 cells were seeded in 60 mm dish and were allowed to grow for 24 h. Compounds chrysin, oroxylin A, methoxy chrysin at 40 μM final concentration as well as TSA (positive control) at 4 μM final concentration were added to the culture media, and the cells were incubated for an additional 24, 48 and 72 h. Cells were harvested with Trypsin-EDTA, fixed with ice-cold 70 % ethanol at 4°C for 30 min, washed with PBS and incubated with 1 mg/ml RNase A solution (Sigma) at 37°C for 30 min. Cells were collected by centrifugation at 2000 rpm for 5 min and further stained with 250 μL of DNA staining solution [10 mg of Propidium Iodide (PI), 0.1 mg of trisodium citrate, and 0.03 mL of Triton X-100 were dissolved in 100 mL of sterile MilliQ water at room temperature for 30 min in the dark]. The DNA contents of 20,000 events were measured by flow cytometer (DAKO CYTOMATION, Beckman Coulter, Brea, CA). Histograms were analyzed using Summit Software.

### Protein extraction and Western blot analysis

5 X 10^5^ A375 cells were seeded in 60 mm dish and were allowed to grow for 24 h. 40 μM concentration of chrysin and 4 μM concentration of TSA were added to the culture media, and the cells were incubated for an additional 24 h. Total cell lysates from cultured A375 cells were obtained by lysing the cells in ice-cold RIPA buffer (1X PBS, 1 % NP-40, 0.5 % sodium deoxycholate and 0.1 % SDS) and containing 100 μg/mL PMSF, 5 μg/mL Aprotinin, 5 μg/mL leupeptin, 5 μg/mL pepstatin and 100 μg/mL NaF. After centrifugation at 12,000 rpm for 10 min, the protein in supernatant was quantified by Bradford method (BIO-RAD) using Multimode varioskan instrument (Thermo-Fischer Scientifics). Fifty micrograms of protein per lane was applied in 12 % SDS-polyacrylamide gel. After electrophoresis, the protein was transferred to polyvinylidine difluoride (PVDF) membrane (Amersham Biosciences). The membrane was blocked at room temperature for 2 h in 1X TBS + 0.1 % Tween20 (TBST) containing 5 % blocking powder (Santacruz). The membrane was washed with TBST for 5 min, and primary antibody was added and incubated at 4°C overnight. P53, p21, p27, cyclin D1, cdk2, cdk4, Bcl-xL and STAT-1, 3, 5a antibodies were purchased from Santacruz and Millipore companies. Survivin, active caspase-3 and β-actin were purchased from Imgenex company. Membranes were washed with TBST three times for 15 min and the blots were visualized with chemiluminescence reagent (Thermo Fischer Scientifics Ltd.). The X-ray films were developed with developer and fixed with fixer solution purchased from Kodak Company.

### HDAC- 8 assay

The HDAC-8 fluorimetric drug discovery kit is based on the unique fluoro de lys HDAC-8 substrate and developer combination. Here the compound was incubated with the fluoro de lys substrate and HDAC-8 (BML-SE 145) for 30 min to observe the inhibitory activity of plant flavonoids (Oroxylin, methoxy-chrysin, chrysin) at a final concentration of 40 μM and known HDAC inhibitor TSA at 4 μM on the HDAC-8 protein. The deacetylation of substrate sensitizes the substrate and developer will produce fluorophore (Enzo Life Sciences USA). The fluorescent readings recorded using Multimode varioskan instrument (Thermo scientific, USA).

### HDAC-1/2 assay

The HDAC-1 and 2 calorimetric assay drug discovery kit is based on the unique Color de lys substrate and developer combination. Here the compound was incubated with the de Color lys substrate and HDAC-1 and 2 (BML-K 1137) for 30 minutes to observe the inhibitory activity of plant flavonoids ( Oroxylin, methoxy-chrysin, chrysin ) at 40 μM and known HDAC inhibitor TSA at 4 μM on the HDAC-1 and 2 proteins. The deacetylation of substrate sensitizes the substrate and developer will produce yellow colour that can be measured by absorption of 405 nm (Enzo Life Sciences USA). The calorimetric readings recorded using Multimode varioskan instrument (Thermo scientific, USA).

### Histone isolation and Western Blotting

The A375 were initially incubated with DMSO, TSA (4 μM) and Chrysin (40 μM) separately in 100 mm dishes with noted concentration for the stipulated time and followed by the washes with cold PBS (2–3 times). The cell lysate was passed through 26 G syringe 10 times and centrifuged at 12,000 g for 20 sec. The pellet was washed briefly with the lysis buffer and again centrifuged. 0.4 N HCl/10 % glycerol was added and incubated in 4°C while shaking. The supernatant was precipitated with 100 % TCA and incubated on ice for 1 h. After centrifugation, histone pellet was washed with acetone/0.02 N HCl, dried and dissolved in water. The histones were run on SDS gel, transferred to nylon membranes and probed overnight at 4°C with rabbit anti-acetyl Histone 3 lysine14, rabbit anti-acetyl Histone 4 lysine 12, rabbit anti-acetyl Histone H4 lysine 16, rabbit anti-dimethyl Histone3 lysine 9 , Histone H3 and Histone H4 (Upstate cell signaling solutions) 1:2000 diluted in 1X TBST and 3 % BSA with 0.02 % Sodium Azide. Appropriate Santa Cruz HRP conjugated secondary antibodies (1:3000) were used. Super Signal West Pico Chemiluminescent Substrate from Pierce was used as per manufacturer’s protocol for developing the blots.

### Indirect Immuno-fluorescence of interphase nuclei

The Colcimid-treated (0.1 μg/ml media for 4–5 hrs) cells were trypsinized and precipitated at 200 *g*, and incubated in 75 mM KCl for 15 min at 37°C and further centrifuged at 100 g. The pellet was dissolved in 5 ml KCl. 300 μl was then mounted on the glass slides at 1000 rpm for 8 min. The slides were fixed in 3.7 % formaldehyde, washed twice with PBS, and treated with PBS containing 0.1 % Triton X-100 and 0.02 % Sodium Azide for 45 min at RT to permeabilize cells. After a wash with PBS, the slides were incubated with the primary antibody overnight at 4°C at 1:200 dilutions with PBT. The slides were washed with PBS for 10 min and incubated in goat serum diluted in PBT (1:50) for 30 min at RT. After PBS wash for 30 min, the slides were counterstained with DAPI and further visualized using confocal microscopy.

### Immunostaining of metaphase chromosomes

We have estimated accumulation of modified histones on the chromosomal arms by indirect Immuno-fluorescence. Briefly, metaphase cell spreads on the slides were incubated for 1 h at 37°C in a humid chamber with serial dilutions with either primary H3 dimethyl Lys-9 (1:50) or Lys-14 acetyl H3 (1:75) Lys 12 acetyl H4 (1:100) antisera, Lys 16 acetyl H4 (1:100) antisera and washed in KCM (120 mM KCl, 20 mM NaCl, 10 mM Tris-Cl- pH 8.0, 0.5 M EDTA, 0.1 % Triton). We had then added Cy3-conjugated, affinity-purified, donkey anti-rabbit IgG antibody (Jackson Immuno-Research) diluted 1:100 in KCM, and incubated the mixture for 30 min at room temperature. Chromosomes were further washed with KCM and fixed in 4 % formaldehyde for 10 min at room temperature. After a wash in sterile water, chromosomes were counterstained with DAPI, mounted the cover slips with anti-fade media (Vectashield) and viewed on a Zeiss Axiophot fluorescence microscope.

### Chromatin Immunoprecipitation Assay (ChIP)

Chromatin immunoprecipitation assay was conducted as described earlier [14] Supplementary protocol). The optimal reaction conditions for PCR were determined for each primer pair. Parameters were denaturation at 95°C for 1 min and annealing at 60°C for 1 min, followed by elongation at 72°C for 1 min. PCR products were analyzed by 2.5 % agarose/ethidium bromide gel electrophoresis. Different primer pairs used for *p21*^*WAF1*^ ChIP analysis (Supplementary materials).

### Immunoprecipitation

A375 Cells were washed twice with PBS, scraped and resuspended in 250 μl of lysis buffer [50 mM Tris (pH-8), 120 mM NaCl, 0.5 % Nonidet P-40, 50 mM NaF, 1 mM sodium orthovanadate, 100 μg of polymethylsulfonyl fluoride/ml, 20 μg of aprotinin/ml, and 10 μg of leupeptin/ml]. The lysates were incubated on ice for 1 h followed by centrifugation at 12,000 rpm for 10 min to remove the insoluble materials. For immunoprecipitations, precleared 0.5 to 1 mg of whole-cell lysates were immunodepleted with p21antibody for 2 h. To this antibody complex, protein A/G agarose (Invitrogen, Inc.) beads were added for 1 h and kept at 4°C in an end-to-end shaker. The beads were washed thrice with lysis buffer without protease inhibitors. 1× Laemmli buffer was added to the beads, samples were boiled and loaded on to SDS-PAGE for western blot analysis using antibodies against STAT-1, 3, 5a.

### Real time PCR Analysis

Total cellular RNA from cells was isolated by Trizol and RNase-Free DNase treatment carried out to remove DNA contaminants. RNA was purified by RNeasy Mini Kit (Qiagen, Germany). Three micrograms of RNA was used for first strand cDNA synthesis using SuperScriptTM (Invitrogen, USA). Real-Time PCR (ABI 7900) was performed. P21 promoter primer sequences for the four different regions were included in the supplementary information.

### Luciferase assay

A375 cells were transfected with wild-type p21-Luc promoter plasmid (1 μg) and CMV-β-galactosidase plasmid (β-gal) (500 ng); mut-p21-Luc promoter plasmid (STAT region is mutated) and CMV-βgal plasmid combinations according to standard transfection protocol. This is followed by compound treatment [chrysin (40 μM), TSA (4 μM)]. The luciferase and β-galactosidase values were determined for each sample separately using Multimode Varioskan Flash (Thermo scientific) instrument. β-gal values were used for normalization.Each experiment was repeated three times and stanadard deviations were derived. Lipofectamine 2000 (Invitrogen) was used as transfection reagent.

### Transcriptional Run-On Analysis

Nuclei were prepared and run-on transcription assays were performed as previously described [14] (supplementary protocol).

### Reverse-Transcription PCR

Total RNA was isolated from the cells treated with chrysin (40 μM) and TSA (4 μM) for 24 h was treated with RNase free DNAse and column purified. Three microgram of RNA was taken for first strand synthesis using superscript reverse transcriptase enzyme (Invitrogen) and PCR was carried using the following primers against **P21 (FP-**5’ atgaaattcaccccctttcc3’ and **RP**-5’ccctaggctgtgctcacttc3’), STAT-1 (**FP-5’** ccgttttcatgacctcctgt 3’ and **RP**-5’tgaatattccccgactgagc3’) and **GAPDH (FP-**5’ acagtcagccgcatcttctt 3’ and **RP**-5’ acaagcttcccgttctcag 3’) was used as internal control.

## Statistical Analysis

Statistical Analysis was performed using the graph pad software to evaluate the significant difference between the control and treated samples. All variables were tested in three independent experiments. The results were reported as mean ± SD. * indicates P <0.05, ** indicates P < 0.01, *** indicates P < 0.001. P values were obtained by comparing compound treated cells with untreated control cells using graph pad software.

## Abbreviations

HDAC, Histone deacetylase; ChIP, Chromatin Immunoprecipitation; H3, Histone H3; H4, Histone H4; CDK, Cyclin dependent kinase; TSA, Trichostatin A; FACS, Fluorescence activated cell sorter; H3ack14, Histone H3 acetylated at lysine14; H4ack12, Histone H4 acetylated at lysine12; H4ack16, Histone H4 acetylated at lysine16; H3me2k9, Histone H3 dimethylated at lysine 9; STAT, Signal transducer and activator of transcription; HDAC-8, Histone deacetylase-8.

## Competing interests

The authors declare that they have no competing interests.

## Author contributions

KSB, JMR, AKT, and JSY designed and synthesized the chemicals. MJR, SNCVLP, TLR and AK conducted the molecular biology and immunofluorescence experiments. MPB and UB designed the biological experiment, analysed the data and wrote the paper. All authors read and approved the final manuscript.

## Pre-publication history

The pre-publication history for this paper can be accessed here:

http://www.biomedcentral.com/1471-2407/12/180/prepub

## Supplementary Material

Additional file 1 Supplementary Data Click here for file
