# Supplementary material for: Plant HDAC inhibitor chrysin arrest cell growth and induce p21WAF1 by altering chromatin of STAT response element in A375 cells
Source: BMC Cancer. 2012 May 16;12:180. doi: 10.1186/1471-2407-12-180 (PMC3407000; doi:10.1186/1471-2407-12-180)
Supplement: Additional file 1 — Supplementary Data [file 1471-2407-12-180-S1.doc]

**Supplementary Online Materials**

**Crude extraction from medicinal plant, *Oroxylum indicum***

After identification of dundilum tree, *Oroxylum indicum,* the stem bark of trees was grinded into powder form and extracted with petroleum ether (60-80^0^C) in a Soxhlet apparatus for 30 hrs. The yellow coloured extract was filtered and purified by column chromatography. The purified filtrate was concentrated under reduced pressure in a rotary flash evaporator to obtain 0.5 gm of the residue. The solid separated from hexane extraction had shown two prominent spots on TLC (solvent system: chloroform: methanol, 90:10 at R_f_ 0.80, 0.75, 0.50) corresponding to oroxylin-A, chrysin.

**Chromatographic separation of the extracts**

The hexane extract (4.0 g) was dissolved in chloroform (20 ml), methanol (5ml) and silica gel (60-120 mesh, 10.0 g) was added. The solvent was removed under vacuum and the powder was transferred to a column of silica gel (60-120 mesh, 300.0 g) set in petroleum ether. The column was eluted successively with chloroform and chloroform: methanol mixture in the order of increasing polarity. Fractions of 100 ml were collected and concentrated.

| Eluting detergent | **Fraction No**. | **Group No**. | *Compound* |
| --- | --- | --- | --- |
| Chloroform : Methanol 99 : 1  Chloroform: Methanol 97 : 3 | 1-20  41-60 | I  III | A  C |

The oroxylin was identified as a bright spot under UV light and a dark spot when sprayed with 5% sulfuric acid in methanol (solvent system: chloroform: methanol 9: 1 at R_f_ 0.80). The fractions were collected to obtain a light yellow solid (0.400 g) compounds using TLC examination of the fraction Group I and III.

**The separation of acetone extract (4.0 g) was performed using the same procedure that isolated a larger light yellow solid referred as compound D.**

| Eluting detergent | **Fraction No**. | **Group No**. | *Compound* |
| --- | --- | --- | --- |
| Chloroform | 1-20 | I | D |

**Purification and characterization of natural flavonoids from** **Oroxylum indicum plant**

The fractions analyzed by the HPLC showed a highest level (more than 99%) of purity. The characterization and structures were established by NMR and ESI-MS analyses. (**Table 1-3**) It was further verified by correlating the obtained spectroscopic results to the earlier data. (Supplementary figure of HPLC graph)

**Supplementary Table 1: NMR, IR and MS data of Chrysin**

Chemical shift Multiplicity Integration ratio Assignment coupling constant

(ppm) (Hz)

12. 77 s 1 4-OH --

10.91 s 1 7-OH --

8.00-7.97 m 2 H-2’, 6’ --

7.55-7.48 m 3 H-3’, 4’, 5’ --

6.89 s 1 H-3 --

6.45 d 1 H-8 *J* = 1.5

6.15 d 1 H-6 *J* = 1.5

**^13^C NMR (75 MHz, DMSO-d6):** δ 181.94, 164.50, 163.22, 161.53, 167.52, 131.09, 130.76, 129.20 (2C), 126.46 (2C), 105.21, 104.03, 99.09, 94.20.

**IR (KBr) ν_max._:** 450, 2925, 1654 cm^-1^.

**Mass**: ESIMS (-Ve): *m/z* 253.1 (M^+^-H).

**HPLC analysis**: t_R_ = 4.71, content = 99.64 and the solvent system, acetonitrile: water, 1:1

**Supplementary Table 2: NMR, IR and MS data for methoxy chrysin**

Chemical shift Multiplicity Integration ratio Assignment coupling constant

(ppm) (Hz)

13.0 s 1 4-OH --

3.96 s 1 7-OMe --

7.96-7.82 m 2 H-2’, 6’ --

7.60-7.44 m 3 H-3’, 4’, 5’ --

6.60 s 1 H-3 --

6.62 d 1 H-8 *J* = 1.5

6.15 d 1 H-6 *J* = 1.5

**^13^C NMR (75 MHz, CDCl_3_):** δ 164.08 (C-2), 105.04 (C-3), 182.88 (C-4), 164.08 (C-5), 93.97 (C-6), 153.31 (C-7), 93.97 (C-8), 153.31(C-9), 105.50 (C-10), 130.96 (C-1′), 126.24 (C-2′), 128.88 (C-3′), 131.26 (C-4′), 128.88 (C-5′), 126.24 (C-6′), 60.63 (OMe).

**IR (KBr) ν_max._:** 450, 2925, 1654 cm^-1^.

**Mass**: EIMS: *m/z* 192 (M^+^).

**Supplementary Table 3: NMR, IR and MS data for oroxylin A**

Chemical shift Multiplicity Integration ratio Assignment coupling constant

(ppm) (Hz)

12.80 s 1 4-OH --

8.18-7.98 m 2 H-2’, 6’ --

7.68-7.48 m 3 H-3’, 4’, 5’ --

6.92 s 1 H-3 --

6.60 s 1 H-8 *------*

3.84 s 3 H-6 *-----*

**^13^C NMR (75 MHz, DMSO-d6):** δ 182.29, 163.27, 157.61, 152.78, 152.59, 132.03, 131.52, 130.76 (2C), 126.41 (2C), 104.68, 104.40, 99.09, 94.44.

**IR (KBr) ν_max._:** 450, 2925, 1622, 1024 cm^-1^.

**Mass**: ESMS: *m/z* 284 (M^+^).

#### Chromatin Immunoprecipitation Assay (ChIP)

#### Chromatin immuno-precipitation assays was conducted. Approximately 2x10^7^ A375 cells were grown in DMEM media containing separately 0.1% DMSO, and treated at 40 µM concentration of chrysin and 4 µM of Trichostatin A (TSA). These A375 cells were cross-linked with formaldehyde to a final concentration of 1% at 37˚C for 10 min. After removing media followed by a quick wash with ice cold PBS containing Protease inhibitors (Boehringer Germany), cells were pelleted and re-suspended in SDS lysis buffer (1% SDS/0 mM EDTA/50 mM Tris·HCl-pH 8.1), and incubated on ice for 10 min. Lysates were sonicated briefly (10-15 sec) and the debris was removed by centrifugation for 10 min at 15,000 X *g* at 4°C. Supernatants were diluted 5-fold in immuno-precipitation buffer (0.01% SDS/1.1% Triton X-100/1.2 mM EDTA/16.7 mM Tris·HCl, pH 8.1/16.7 mM NaCl), and 80 µl of a 50% protein A sepharose slurry containing 25 µg sonicated salmon sperm DNA and 1 mg/ml BSA in TE buffer (10 mM Tris·HCl, pH 8.0/1 mM EDTA) was added and incubated with shaking for 2 hrs at 4°C. After pelleting the protein beads by centrifugation, supernatants were placed in the fresh tubes with 5 µl of anti-rabbit anti- histone H3acK14, H4acK12 antibodies, anti-dimethyl histone H3K9 (Upstate, USA), anti-rabbit polyclonal STAT-1 (Santa Cruz, USA) antibodies and normal rabbit serum independently and incubated overnight at 4°C. Protein A sepharose slurry (60 µl) was added to the tube and mixed by shaking for1 h at 4°C. After thorough wash for at least 5 times (5 min each), Protein A complexes were eluted twice with 250 µl of elution buffer (1% SDS/0.1 M NaHCO_3_) for 15 min at room temperature. Further 20 µl of 5 M NaCl was added to the solution by incubating at 65°C for 4 h. Proteinase K was then added to the samples at a final concentration of 10 mM, 40 mM, and 0.04 µg/µl, respectively, and incubated further at 45°C for 1 h. DNA fragments (both immunoprecipitation samples and Input) were recovered by phenol/chloroform extraction and ethanol precipitated for analysing by PCR. *p21^WAF-1^* promoter primers were used to carry out PCR from DNA isolated from ChIP experiment.

The optimal reaction conditions for PCR were determined for each primer pair. Parameters were denatured at 95°C for 1 min and annealed at 60°C for 1 min, followed by elongation at 72°C for 1 min. PCR products were analyzed by 2.0% agarose/ethidium bromide gel electrophoresis. The primer pairs used:

Forward primer 5’-CGT GGT GGT GGT GAG CTA GA-3’ (*p21^WAF1^* primer 1),

Reverse primer 5’-CTG TCT GCA CCT TCG CTC CT-3’ (*p21^WAF1^* primer 1),

Forward primer F5’-GGT GTC TAG GTG CTC CAG GT-3’ (*p21^WAF1^* primer 2),

Reverse primer 5’-GCA CTC TCC AGG AGG ACA CA-3’ (*p21^WAF1^* primer 2),

Forward primer 5’-ACC AAC GCA GGC GAG GGA CT-3’ (*p21^WAF1^* primer 3),

Reverse primer 5’-CCG GCT CCA CAA GGA ACT GA-3’ (*p21^WAF1^* primer 3),

Forward primer 5’-ACC GGC TGG CCT GCT GGA ACT-3’ (*p21^WAF1^* primer 4),

Reverse primer 5’-TCT GCC GCC GCT CTC TCA CCT-3’ (*p21^WAF1^* primer 4),

Forward primer 5’-AGG AAT CCC TGG TCA CGC TC-3’ (*p21^WAF1^* Input 1),

Reverse primer 5’-GTG GTG GAC ACA GTG GCG TA-3’ (*p21^WAF1^* Input 1).

**Quantitation of fluorescence intensity from interphase nuclei**

Once immuno-staining of the interphase nuclei was completed, the nuclei images from the Cy5 excitation channel were analyzed for measuring the fluorescence in each nuclei by determining the gray scale level using Metamorph version 4.6 (Universal imaging, USA) software. Gary scale, which is defined as the brightness of pixels in a digitized image, is an eight-bit digital signal with 256 possible values ranging from 256 (white) to 0 (black). Mean gray scale values are equal to the total gray scale values per number of pixels. For mean gray value, total gray scale values of interphase nuclei were calculated by dividing the total pixel area of each interphase nuclei image.

The nuclei prepared without primary antibodies served for background determination of each antibody used. The mean value of the background images for each antibody was calculated and then individually subtracted from each interphase nucleus. The changes in fluorescence intensity by a particular compound and DMSO treatment were estimated. The nuclei incubated in each compound and DMSO was compared. The relative values allow to determine the net intensity of fluorescent value and plotted in a bar diagram.

**Transcriptional Run-On Analysis**

Nuclei were prepared and run-on transcription assays were performed. For the run-on assay, 40 µl of run-on buffer was added to the (80 mM Tris·HCl, pH 7.5/10 mM MgCl_2_/1 mg/ml heparin/0.6% (wt/vol) Sarkosyl/350 mM (NH_4_)_2_SO_4_/400 µM ATP/400 µM GTP/400 µM UTP/250 µCi [α^32^P]-CTP) 20 µl of packed nuclei and the entire mixture were incubated at 37°C for 30 min. After incubation, 10 µg of tRNA, 175 µl of 20 mM Tris·HCl containing 10 mM CaCl_2_, 25 µl of a solution containing 1 mg/ml proteinase K and DNase I were further added and incubated at 37°C for 30 min and the reaction was stopped by addition of 25 µl of 10% (w/v) SDS and 25 µl of 0.2 M EDTA. The labeled RNA was then extracted with phenol/chloroform/isoamyl alcohol (25:24:1). Equal amounts of ^32^P-labeled RNA, (approximately 5 × 10^7^ cpm) were then hybridized to nylon membranes at 60˚C containing 5 µg of *p21^WAF1^* and ß-actin cDNA. After washing the filters were exposed to X-ray film at −70°C for 2-10 days with intensifying screen. The relative rate of transcription was determined by phosphorimager.

**Figure S1.** Chemical structure, HPLC purification of three flavonoids, chrysin, methoxy-chrysin and oroxylin. Area% of each peak represents % of purity.

**Figure S2. ^1^**H NMR spectrum of chrysin

**Figure S3** ^13^C- NMR spectrum of chrysin
